# Physical activities awareness and practice among the healthcare professions students in Saudi Arabia

**DOI:** 10.3389/fpubh.2025.1624914

**Published:** 2025-06-30

**Authors:** Eidan M. Al Zahrani, Salah H. Elsafi, Abdulwahab H. Alharbi, Wadha S. Alotaibi, Aqeelah A. Alyahya, Sarah A. Alqahtani, Shatha M. Al Thabet, Shrooq O. Al Harbi, Ahmed A. Adam, Abdallah A. Al Shehri

**Affiliations:** ^1^Physical Therapy Department, Prince Sultan Military College of Health Sciences, Dhahran, Saudi Arabia; ^2^Clinical Laboratory Sciences Department, Prince Sultan Military College of Health Sciences, Dhahran, Saudi Arabia; ^3^Basic Medical Unit, Prince Sultan Military College of Health Sciences, Dhahran, Saudi Arabia; ^4^Vice Deanship of Development and Quality, Prince Sultan Military College of Health Sciences, Dhahran, Saudi Arabia

**Keywords:** attitude, healthcare, knowledge, practice, Saudi Arabia, students

## Abstract

**Objective:**

This study aimed to measure and correlate the level of awareness, attitude, and practice of physical activity among healthcare professional students with various demographical variables.

**Methods:**

This cross-sectional study used a structured online questionnaire. A heterogeneous purposive sample, according to the study’s objectives, of health professional students belonging to 15 universities in Saudi Arabia from February to May 2024.

**Results:**

The overall knowledge, positive attitude, and practice scores of the participants towards the physical activity guidelines were moderate as represented by 68.97%, 47.93 %, and 51.90%, respectively. Unlike knowledge and attitude, practice varies among all demographic features. Participants identified walking as the most common form of exercise, 60.64%, followed by moderate-intensity activity,16.15%, and vigorous-intensity activity, 9.17%, while 14.04% reported no practice. Barriers to practice PA included lack of time, 55%, lack of interest, 30%, and illness/injury, 6%. There was a significant relationship between the mean practice score and both knowledge and attitude scores (*p* = 0.001 and 0.003, respectively).

**Conclusion:**

The study indicated a relatively moderate awareness and practice of physical activity that varied according to the demographic features due to regional differences in educational systems, cultures, and socioeconomic classes. The study highlighted the possibility of focused interventions to close the knowledge-behavior gap and encourage active lives in this community.

## 1 Background

Physical activity (PA) is any movement made by the body’s skeletal muscles that involves an energy expenditure measured by calories. Exercise is a subset of PA that is planned, structured, and repetitious to maintain or improve PA (1). Both adults and children’s health is directly impacted by PA (2). The minimum amount of PA for adults is aerobic activity of 2 h 30 min per week of moderate-intensity or 1 h 15 min of high intensity (3).

According to epidemiological data, physical inactivity raises the occurrence of at least 17 harmful conditions, most of which are chronic diseases or risk factors for chronic diseases (4). Physical inactivity is a global public health concern, affecting most of the world’s population. Exercise is crucial for promoting good health and preventing disease. On the other hand, physical inactivity is a risk factor for various chronic diseases (5). The most common causes of premature death are stress, obesity, and physical inactivity. Sedentary lifestyles double the risk of lipid disorders, obesity, hypertension, diabetes, and cardiovascular disease, thus increasing mortality.

Many factors influence PA for health, including environmental, socio economic, cultural, and lifestyle. For example, individuals of poor socioeconomic positions are likely to have lower PA levels since they have little access to parks and local walkability (6).

Several guidelines for physical activity have been developed to inform people about the minimal quantity of PA that is generally required to establish cardiorespiratory health and improve health (7). The benefits of aerobic and resistance training on cognition and health are mediated by a number of processes, including improved neurogenesis, decreased oxidative stress and inflammation as well as improved cerebral blood flow and oxygen delivery, and favorable hormonal changes (8, 9).

Information on peoples’ awareness and attitudes toward PA is essential to promote practice and identify the PA barriers. This is because awareness dramatically influences an individual’s positive attitude thus, improving the practice.

It has been documented that insufficient knowledge is considered to be a significant contributing factor responsible for PA practice (10). Lack of knowledge about PA guidelines by the healthcare profession student, who should be the most knowledgeable, has previously been noticed as one of the main factors that may contribute to a sedentary lifestyle. Despite the apparent rise in awareness of PA requirements in recent years, it has been reported that demographic differences persist (10). Knowledgeable medical students are more likely to advocate PA and to help in bringing about an increased awareness and thus improve the attitudes of others toward PA. A previous study reported a good knowledge and positive attitudes toward PA among the healthcare profession students (11). The prevalence of PA was 69.9% among healthcare students (12). Another study showed that about 50% of the students were physically active, while 20% relied solely on short walks due to time constraints, limited activity hours, or sedentary habits (13). Several studies have shown that university students are becoming physically inactive (14, 15).

Students’ PA levels are frequently influenced by their academic schedules, social and familial obligations, and lack of leisure time. Few studies have been conducted on the attitude toward PA among university students of Saudi Arabia compared with their global counterparts. Moreover, few studies have assessed awareness and knowledge of PA guidelines, mainly conducted on the general population (16, 17).

A recent study included 507 surveys across 163 countries and territories. Indicated that nearly one third (31%) of the world’s adult population are physically inactive (18). A previous study has shown that more than half of college students need to be more active, and their levels of PA are significantly associated with some demographical characteristics of the students but not their knowledge of the recommended guidelines (19). Based on the preceding facts, and because many students feel pressure when engaging in academic activities, leaving little time for PA, it was essential to assess the current level of physical activity among university students. Despite healthcare profession’s students being future health promoters, limited research has examined the knowledge-attitude-practice relationship in this specific population within the Saudi cultural context. This study aimed to: (1) assess knowledge, attitudes, and practices regarding physical activity among Saudi healthcare students, (2) examine demographic correlates of these variables, and (3) investigate the relationships between knowledge, attitudes, and practice behaviors.

## 2 Materials and methods

### 2.1 Study design and ethical consideration

This cross-sectional study used a structured questionnaire prepared by the research group based on the study’s objectives. This study was authorized by the Ethics Review Board at Prince Sultan Military College of Health Sciences in Dhahran (IRB Number IRB-2024-CLS-28). The questionnaire was anonymized, and the participants were not required to provide personal information. The participants were asked to electronically sign a brief agreement outlining the study’s goals and guaranteeing the privacy of their personal information as a condition of participating in it. All participants signed a written informed consent form electronically.

### 2.2 Instruments

A well-structured and pretested questionnaire was developed for this study according to the objectives. The study group produced the questionnaire after conducting an extensive review of the existing literature. There were 25 closed- and open-ended questions on the questionnaire. The first section included six demographic variables such as age, gender, region, academic major, study year, body appearance, and fitness level. The second section included seven knowledge multiple-choice questions about the types and usefulness of physical exercise. The third section consists of three statements on a five-point Likert scale ranging from strongly agree to strongly disagree about the participants’ attitude toward physical exercise. The fourth section included three questions related to the participant’s practice of physical exercise. The last section of the questionnaire included six questions about the barriers to practicing PA. A team of experts evaluated the questions before administering a pilot study on 87 participants who were not part of the study. The pilot study data were examined for the questionnaire’s internal consistency reliability using Cronbach’s alpha, which yielded a coefficient value of 0.83, indicating a good reliability.

### 2.3 Participants

A heterogeneous purposive sample of Medicine, nursing, and applied medical sciences healthcare students at all universities countrywide. This sampling method was specifically chosen to include a diverse range of participants from various healthcare professions’ students at various Saudi universities, which aligns with the study’s objectives of investigating how demographic variable, such as educational background and regional differences are related to physical activity awareness, attitude, and practice. We included a maximum number of cases that represent the entire characteristics included in the objectives of the study.

The survey included the healthcare profession’s students’ of 15 universities belonging to the five main regions of Saudi Arabia: The Central, Western, Eastern, Northern, and Southern Regions. The questionnaire was distributed to participants through a web link they could access on several social media sites. It was made available from February 20 until May 7, 2024.

### 2.4 Statistical analysis

Two score were allocated to each of the seven knowledge multiple-choice questions, for each correct answer, making a total of 14 points and the percentage of the correct responds were calculated. Wrong answers received no scores. The participants’ attitudes were assessed through the use of five-point Likert scale rating questions: strongly agree (5), agree (4), neutral (3), disagree (2), and strongly disagree (1). The average attitude score was calculated out of 15 for the three questions. The average percentages of physical activity practice were calculated for the positive responses to the three related questions.

The results were analyzed using SPSS software version 30.0 (SPSS, Chicago, IL, USA). We employed a bivariate correlation between knowledge, attitude, and practice and one-way ANOVA to assess for significant variations related to demographic variables. Statistical significance was set at *p* < 0.05 for all analyses.

## 3 Results

### 3.1 Participant characteristics

Out of 1,288 responders, 1,189 (92.3%) successfully completed the questionnaire and included in this study. Table 1 shows the demographical characteristics of the 1,189 participants. Most participants, 75.3%, were aged 18–23. Female students represented 69.4%. The participants belonged to five regions of Saudi Arabia. Most students were from applied health sciences, making up 66.8% of the total in various study years (first to fifth). 58.3% of people were of normal weight, whereas just 4.0% of people were obese.

**Table 1:** Demographical characteristics of the healthcare professions students (*n* = 1,189).

Group		Frequency	Percentage
Age in years	18–20	419	35.2
	21–23	477	40.1
	>23	293	24.6
Gender	Male	364	30.6
	Female	825	69.4
Region	Eastern region	356	29.9
	Western region	156	13.1
	Central region	347	29.2
	Northern region	65	5.5
	Southern region	265	22.3
Academic major	Medicine	86	7.2
	Nursing	309	26
	Applied health sciences	794	66.8
Level	First-year	210	17.7
	Second year	294	24.7
	Third year	243	20.4
	Fourth-year	215	18.1
	Internship	277	19.1
Body appearance	Underweight	205	17.2
	Normal weight	693	58.3
	Overweight	244	20.5
	Obese	47	4.0

### 3.2 Knowledge, attitude, and practice of physical activity

Table 2 displays the participants’ mean knowledge, attitude, and practice scores according to the various demographical features. The overall knowledge score of 68.97%. There were significant differences in the mean knowledge score across the multiple regions and body appearances, with *p*-values of 0.005 and 0.002, respectively. The participants from the Central Region showed the highest average knowledge score of 71.13%. Overweighed students demonstrated the highest mean knowledge score of 70.05%. Nevertheless, the differences in the knowledge scores among different age groups, genders, academic majors, or levels were insignificant. The overall positive attitude score was 47.93%. There was evidence supporting the variation in the average attitude scores among various study majors (*p* = 0.005). Medicine students had the highest mean attitude scores at 53.10%, followed by applied health sciences students at 48.39%.

**Table 2:** Percentage knowledge, attitude, and practice mean scores and the 95% CI of the healthcare professions students by toward PA by demographical variables (*n* = 1,189).

Demographic characteristics		*N* (%)	Knowledge		Attitude		Practice	
			% Mean (95% CI)	*p*-value	% Mean (95% CI)	*p*-value	% Mean (95% CI)	*p*-value
Age group	18–20	419 (35.2)	68.90 (67.23–70.57)	0.6	48.75 (46.80–50.67)	0.61	52.33 (49.77–54.89)	0.029
	21–23	477 (40.1)	68.45 (66.69–70.21)		47.46 (45.51–49.41)		49.62 (47.05–52.19)	
	>23	293 (24.6)	69.90 (67.43–72.37)		47.55 (45.27–49.83)		54.98 (51.97–57.99)	
Gender	Male	364 (30.6)	69.29 (66.94–71.64)	0.72	49.68 (47.37–51.99)	0.06	60.52 (57.78–63.26)	<0.001
	Female	825 (69.4)	68.82 (67.62–70.02)		47.16 (45.80–48.52)		48.09 (46.25–49.93)	
Region	Eastern	356 (29.9)	70.20 (68.44–71.96)	0.005	46.89 (44.69–49.09)	0.1	49.46 (46.51–52.41)	0.044
	Western	156 (13.1)	65.19 (61.64–68.73)		46.85 (43.53–50.17)		49.87 (45.50–54.24)	
	Central	347 (29.2)	71.13 (69.23–73.03)		50.52 (48.40–52.64)		51.81 (48.99–54.63)	
	Northern	65 (5.5)	66.42 (60.85–71.99)		47.89 (42.67–53.09)		54.15 (47.73–60.57)	
	Southern	265 (22.3)	67.32 (64.83–69.81)		46.76 (44.28–49.24)		55.92 (52.70–59.14)	
Academic major	Medicine	86 (7.2)	64.70 (59.28–70.12)	0.69	53.10 (48.46–57.74)	0.005	50.69 (44.25–57.13)	0.836
	Nursing	309 (26.0)	68.43 (66.22–70.64)		45.32 (44.28–49.24)		52.55 (49.50–55.60)	
	App. Hlth. Sci.	794 (66.8)	69.63 (68.28–70.97)		48.39 (46.94–49.84)		51.77 (49.88–53.66)	
Level	First-Year	210 (17.7)	70.02 (67.81–72.23)	0.36	48.79 (45.82–51.75)	0.06	50.85 (47.28–54.41)	0.002
	Second Year	294 (24.7)	67.34 (65.13–69.55)		47.34 (45.03–49.65)		52.27 (49.32–55.22)	
	Third Year	243 (20.4)	69.25 (66.55–71.95)		45.81 (43.22–48.40)		51.76 (48.31–55.21)	
	Fourth Year	215 (18.1)	68.29 (65.88–70.70)		46.94 (44.08–49.80)		46.79 (42.85–50.72)	
	Internship	47 (19.1)	70.43 (64.47–76.39)		51.13 (45.41–56.84)		57.35 (49.4–65.29)	
Body appearance	Underweight	205 (17.2)	66.16 (63.67–68.65)	0.002	49.26 (46.50–52.02)	0.33	47.70 (43.61–51.79)	<0.001
	Normal	693 (58.3)	69.96 (68.58–71.34)		48.00 (46.44–49.56)		56.07 (54.12–58.02)	
	Overweight	244 (20.5)	70.05 (67.76–72.34)		46.14 (43.7–48.57)		47.33 (44.04–50.62)	
	Obese	47 (4.0)	60.82 (51.00–70.64)		50.49 (42.79–58.19)		32.34 (24.82–39.86)	

App. Hlth. Sci., Applied Health Sciences; CI, Confidence Interval.

Table 2 also illustrates a significant variation in the average practice scores across age groups, gender, region, level, and body appearance. The overall practice score was 51.90%. The older age group of >23 showed the highest average practice score of 54.98%. Furthermore, regarding gender, the mean practice score for male students was 60.52%. The Southern Region had the highest average practice scores among all the regions, with a score of 55.92%. The academic year showed variations in average practice scores, with a higher mean of 57.35% for individuals who were interns. Individuals with an average weight had a mean practice score of 56.07%.

### 3.3 The features of individuals who exercise vigorously and those who do not

Table 3 illustrates the frequency of vigorous exercise among the participants. Significant differences in gender, body appearance, and current fitness level were associated with vigorous exercise practice. Males exhibit a higher level of physical activity than females, accounting for 90.7% of the participants. Most students who perform vigorous exercise maintain a normal weight (89.5%), and their current level of physical fitness is perfect. There was no significant correlation between vigorous exercise and the other demographic characteristics.

**Table 3:** The features of the healthcare professions students who exercise vigorously and those who do not (*n* = 1,189).

Demographic characters		*N*	Number of practicing students and (%)	Number of nom-practicing students and (%)	*p*-value
Gender	Male	364	330 (90.7)	34 (9.3)	<0.001
	Female	825	692 (83.9)	133 (16.1)	
Age	18–20	419	365 (87.1)	54 (12.9)	0.11
	21–23	477	398 (83.4)	79 (16.6)	
	>23	293	259 (88.4)	34 (11.6)	
Region	Eastern	356	297 (83.4)	59 (16.6)	0.329
	Western	156	134 (85.9)	22 (14.1)	
	Central	347	297 (85.6)	50 (14.4)	
	Northern	65	58 (89.2)	7 (10.8)	
	Southern	265	236 (89.1)	29 (10.9)	
Academic major	Medicine	86	70 (81.4)	16 (18.6)	0.446
	Nursing	309	266 (86.1)	43 (13.9)	
	App. Hlth. Sci.	794	686 (86.4)	108 (13.6)	
	First-year	210	177 (84.3)	33 (15.7)	
	Second-year	294	262 (89.1)	32 (10.9)	
	Third-year	243	207 (85.2)	36 (14.8)	
	Fourth-year	215	174 (80.9)	41 (19.1)	
Level	Internship	227	202 (89.0)	25 (11.0)	0.055
Body appearance	Underweight	205	169 (82.4)	36 (17.6)	<0.001
	Normal weight	693	620 (89.5)	73 (10.5)	
	Overweight	244	207 (84.8)	37 (15.2)	
	Obese	47	26 (55.3)	21 (44.7)	
Current level of fitness	Perfect	227	223 (98.2)	4 (1.8)	<0.01
	Good	345	325 (94.2)	20 (5.8)	
	Average	456	391 (85.7)	65 (14.3)	
	Poor	135	71 (52.6)	64 (47.4)	
	Unfit	26	12 (46.2)	14 (53.8)	

### 3.4 Characteristics of physical activity practice

Table 4 reported the participants’ responses to various questions about physical activity practice among the students in the healthcare profession. Participants identified walking as the most common form of exercise, with an average rate of 60.64%. Subsequently, there is a frequency of moderate-intensity activity at 16.15%, followed by vigorous-intensity activity at 9.17%. 14.04% of individuals reported no practice of physical activity. Table 4 also shows the frequency of vigorous physical activity among the participants. 31.3% of the participant’s report exercising several times per week, while 19.1% indicate exercising once weekly. However, 20.4% of the participants reported not being involved in intense exercise. Table 4 also shows the various exercise barriers reported by the participants. The table indicates that 16.70% of participants report having medical restrictions that prevent them from exercising. A notable proportion of participants, 39.20%, reported engaging with exergames, suggesting an inclination toward or prior interest in this type of physical activity.

**Table 4:** Reported responses to various questions about physical activity practice among the healthcare professions students (*n* = 1,189).

How do you classify your exercise activity?	Frequency (%)
Walking activity	721 (60.64)
Moderate-intensity activity (e.g., gentle swimming)	192 (16.15)
Vigorous intensity activity (e.g., cycling)	109 (9.17)
I do not exercise	167 (14.04)
How often do you vigorously exercise?	
Every day	131 (11.00)
Several times per week	372 (31.30)
Several times per month	13 (1.10)
Once per week	227 (19.10)
Less often	203 (17.10)
I do not practice	243 (20.40)
Do you have any medical limitations preventing you from exercise?	
Yes	199 (16.70)
No	990 (83.30)
If your participation is lower than you would like it to be, what are the reasons?	
Lack of time	652 (55.00)
Lack of interest	352 (30.00)
Illness/Injury	74 (6.00)
Others: laziness, lack of facility, lack of accompany	47 (4.00)
More than one reasons	64 (5.00)
Did you try to play with exergames?	
Yes	466 (39.20)
No	723 (60.80)

### 3.5 Correlation between knowledge, attitude, and practice

Table 5 shows the Pearson correlation coefficient between the mean knowledge, attitude, and practice score. The relationship between the knowledge mean score of 68.97% and the attitude mean score of 47.93% suggested a negligible association, with a correlation coefficient (*r*) of 0.042. However, this correlation was not statistically significant, as the (*p*-value = 0.15).

**Table 5:** Pearson correlation analysis between knowledge, attitude, and practice scores of physical activity among the healthcare professions students (*n* = 1,189).

Measured variable	Mean (95% CI)	Pearson correlation	*p*-value
Knowledge score	68.97 (67.87–70.07)	0.042	0.15
Attitude score	47.93 (46.75–49.11)		
Practice score	51.90 (50.34–53.46)	0.087	0.003
Attitude score	47.93 (46.75–49.11)		
Practice score	51.90 (50.34–53.46)	0.104	0.001
Knowledge score	68.97 (67.87–70.07)		

Table 5 also illustrates the relationship between the total score for practice and the total score for attitude. A Pearson correlation coefficient (*r*) of 0.087 (*p* = 0.003) indicated a slight positive relationship between the practice mean and attitude mean scores. Our analysis has determined a notable correlation between the two variables, suggesting that students’ attitudes show improvement and their practice scores experience a slight increase. The table displays the correlation between the mean score of practice and the mean knowledge score, along with the mean of 51.9 and 68.97%, respectively. The Pearson correlation coefficient yielded a *p*-value of 0.001. There was a significant relationship between the mean practice score and the knowledge total score, with a correlation coefficient of *r* = 0.104.

## 4 Discussion

This study provides key insights into the knowledge, attitudes, and practices of PA among healthcare undergraduates in Saudi Arabia. The overall knowledge score of 68.97% indicates a relatively moderate awareness of PA. While knowledge scores averaged 81% in the U.S., the current awareness in Saudi Arabia of 68.97% represented a moderate rate that requires targeted educational interventions (20, 21). Moreover, another study has shown that most (67.6%) of people in the United States were aware of PA recommendations (16). Knowledge of critical PA was moderate (75%) and varied between various demographical characteristics in Australia (22). The average knowledge score of the students on physical activity in Nepal was 71.3% (23).

The knowledge scores exhibited significant variation based on region. Various factors could cause this disparity, most of which are related to regional differences in educational systems and cultures similar to what has been reported as reported before (24). The study showed that male students demonstrated slightly higher knowledge scores than female ones. Similarly, previous research has shown similar results with no gender difference in knowledge (25). The average knowledge score of participants of different ages slightly increased in older students. Similarly, previous study results showed good knowledge among older people (26). In contrast, another study shows that older adults possessed poorer PA knowledge than their younger counterparts (27). The participants from the medicine colleges have the lowest knowledge score on PA, while the applied health sciences colleges have a high knowledge. These discrepancies might be caused by variations in the curriculum’s emphasis, availability of resources for health education, or the dominant beliefs and practices in specific academic or geographical contexts.

The average attitude score was 47.93%. Males showed a slightly more positive attitude than females. Women’s participation in sports substantially correlated with socioeconomic class, health-relevant attitudes, including internal and external locus of control, and health markers. Another study reported an average positive attitude score toward physical activities of 64.3% among undergraduate medical students in Nepal (24). A previous study has shown that medical students’ attitudes regarding PA were positive and did not differ from those of male and female students (28). The study found that the average attitude score of participants increased among 18–20 aged students. The results show that the average attitude score of participants in different regions varied slightly. The average attitude score of participants across the study majors was statistically significant. Medicine students have higher attitude scores in PA, while nursing students have lower scores.

The study shows a worrying trend in PA habits, with a sizable fraction reporting no intense exercise and a minority regularly engaging in vigorous-intensity activities. This trend is consistent with earlier research showing startlingly low PA levels among college students (29). About 62% of participants in a study on female university students failed to achieve the WHO standard of 75 min per week of strenuous activity, and 70% of participants failed to meet the WHO recommended of 150 min per week of moderate activity (16).

Walking is the most popular type of exercise, which is essential since it is accessible and low-impact, making it a viable option for many participants. Across all participants, the average practice was 51.9%. A sizeable fraction of 14.04% acknowledged never exercising, compared to 3.9% reported before (30). There was a statistically significant difference in practice by gender, with men exhibiting somewhat more than women. The current study also revealed that women were less likely than men to exercise. A prior study found a substantial difference between males and females in total physical activity and vigorous-intensity physical exercise (20).

Another study determined that 68.4% of men and 48.4% of women university students reported practicing PA (2, 31). Gender inequalities can be explained by a variety of reasons, including weariness and exhaustion, a lack of social support, culturally imposed gender norms and behavioral expectations that favor women staying at home, and limited facilities for women’s sports. Similarly, research has noticed that boys from several countries participate in more PA than females, likely contributing to their better PA assessment scores (32). Also, another study shows that boys have more substantial incentives to participate in PA, whereas girls exhibit more obstacles (33). The two most often cited barriers were time constraints and a lack of encouragement from teachers, classmates, and families. The main barriers that prevent students from participating in PA most frequently are a lack of time and poor facilities (20).

Illness and injuries were less reported by the participants. However, implementing customized fitness programs, and integrating subjective and objective training load evaluations could all help to lower injury rates, increase recruit preparedness (34). The absence of companionship and insufficient time contributed to university students’ avoidance of PA (24). The study represents a high percentage of PA practice among all age groups. The age group over 23 exhibited the highest proportion, accounting for 54.98%. The nursing department students demonstrated a higher level of proficiency (52.55%) compared to the medicine students, who showed a lower level of proficiency (50.69%). The study showed that the students who were in perfect present fitness (71.45%) or had an average weight (56.07%) were more physically active. Conversely, those who were obese (32.34%) or unfit to the current standard of fitness (13.46%) were less likely to engage in PA. Being overweight during childhood and adolescence is a typical cause of a sedentary lifestyle among students (35). It is important to note that Saudi Arabian institutions’ particular cultural and environmental settings may present unique obstacles and opportunities for promoting physical exercise.

The observed differences in knowledge, attitude, and practice among subgroups emphasize how crucial it is to customize treatments to meet specific student populations’ needs and obstacles.

For example, students from different academic majors or areas with lower knowledge scores could benefit from more intensive health education programs. People with low socioeconomic positions probably engage in less physical exercise since fewer parks and walkable neighborhoods exist in their communities (6).

There was a significant relationship between the mean practice score and both knowledge and attitude scores. These findings were in contrast to those previously determined, which showed that there was no correlation between the amount of knowledge regarding PA and its practice (36). We found no correlation between knowledge and attitude.

The Theory of Planned Behavior that links beliefs to behavior, may provide a good model to explain the relationship between the healthcare profession’s students’ knowledge, attitude, and practice of PA (38). According to the theory, individual’s behavior is influenced by intention, which in turn is governed by attitude, perception of social pressure (subjective norms), and belief in their ability to perform a specific behavior (perceived behavioral control). In this study, the moderate level of PA attitude (47.93%) together with its significant correlation with practice may suggests that the students’ intentions could be hindered by their perceptions of social expectations or their own ability to be engaged in physical activates. The reported barriers, such as lack of time and or interest, may constrain the translation of positive knowledge into consistent action thus lowering their ability to practice PA.

This miscorrelation between attitude and knowledge is consistent with other research (30, 35). Interestingly, the study found no links between participants’ attitudes toward physical exercise and demographic variables. This conclusion contradicts some earlier research (27, 35) that found opinions differing depending on demographic variables. It is crucial to remember that attitudes are complicated concepts that are impacted by a wide range of variables. Therefore, the study’s lack of significant correlations could be unique to the environment and population it looked at.

Overall, the study’s findings highlight the urgent need for focused interventions and approaches to close the knowledge–behavior gap and remove the PA obstacles that college students in healthcare professions programs face. Some possible approaches are to incorporate opportunities for PA into the curriculum to encourage students to engage in recreational activities on campus and to provide easily accessible and reasonably priced exercise facilities that meet their various needs and preferences. Furthermore, programs focusing on time management abilities and social support systems may enable students to prioritize physical exercise above academic obligations. Despite the good knowledge and positive attitudes toward.

PA, its prevalence was estimated to be 51.90% in this study and, 69.9% by another study among healthcare students in Saudi Arabia (13, 37). A previous study revealed that 55.7% of the students performed vigorous-intensity sports, fitness, or recreational activities (36). Overall, 58.0% of the students we physically inactive, and only 13.4% performed vigorous physical activity, 14.8% moderate-intensity PA, and 29.9% walking activities (36). A study in Saudi Arabia showed that the most common type of PA among healthcare students was walking (51.7%), followed by bodybuilding (25%) and running at 24.4% (10). The top reported barrier to PA among inactive students was time limitations (51.3%).

Our limitations are typical to the other studies using a cross-sectional design and self-reported data. The results may not be as broadly applicable as we used a heterogeneous purposive sample technique. Confounding variables that are not measured.

## 5 Conclusion

This study highlights a moderate PA knowledge and practices among healthcare students in Saudi Arabia, with notable disparities across gender, age, and academic majors. Targeted interventions, including curriculum integration and improved access to facilities, are essential to bring a higher knowledge–behavior and practice levels and promote healthier lifestyles. The disparity between participants’ reported PA attitude and knowledge levels is one of the study’s noteworthy results. Universities could incorporate PA modules into existing health courses and create on-campus programs emphasizing time management skills to help students balance academics and exercise. Future research using a longitudinal study design and intervention trials are needed for better understanding of the suboptimal level of the university students’ practice of PA despite the good knowledge.

## Data Availability

The raw data supporting the conclusions of this article will be made available by the authors without undue reservation.
